# Supplementary material for: Compression theory for inhomogeneous systems
Source: Nat Commun. 2024 Nov 25;15:10214. doi: 10.1038/s41467-024-54341-8 (PMC11589113; doi:10.1038/s41467-024-54341-8)
Supplement: Supplementary file 1 — Supplementary Information [file 41467_2024_54341_MOESM1_ESM.pdf]

Supplementary information: Compression theory for inhomogeneous systems

Doruk Efe Gökmen,[1,2,3,4,*] Sounak Biswas,[5] Sebastian D. Huber,[1]
Zohar Ringel,[6] Felix Flicker,[7,†] and Maciej Koch-Janusz[8,2,9,‡]

[1] *Institute for Theoretical Physics, ETH Zurich, 8093 Zurich, Switzerland*
[2] *James Franck Institute, The University of Chicago, Chicago, IL 60637, USA*
[3] *Department of Statistics, The University of Chicago, Chicago, IL 60637, USA*
[4] *National Institute for Theory and Mathematics in Biology, Chicago, IL 60611, USA*
[5] *Institut für Theoretische Physik und Astrophysik, Universität Würzburg, 97074 Würzburg, Germany*
[6] *Racah Institute of Physics, Hebrew University, Jerusalem, 9190401, Israel*
[7] *School of Physics, Tyndall Avenue, Bristol, BS8 1TL, United Kingdom*
[8] *Department of Physics, University of Zürich, 8057 Zürich, Switzerland*
[9] *Haiqu, Inc., 95 Third Street, San Francisco, California 94103, USA*

I. THE ODD SCALE TRANSFORMATIONS OF DIMER COVERINGS ON THE AB TILING

Our analysis of the coarse graining transformations of the dimer model on the AB tiling *did not* provide evidence for a discrete scale invariant description in terms of super-dimer variables under all rescalings, but only for *even* order ones (*i.e.* under deflations σ^{-2k}, for $k \in \mathbb{N}_0$). This is in contrast to the AB tiling itself (*i.e.* just the AB quasilattice), which is invariant under any order of deflation.

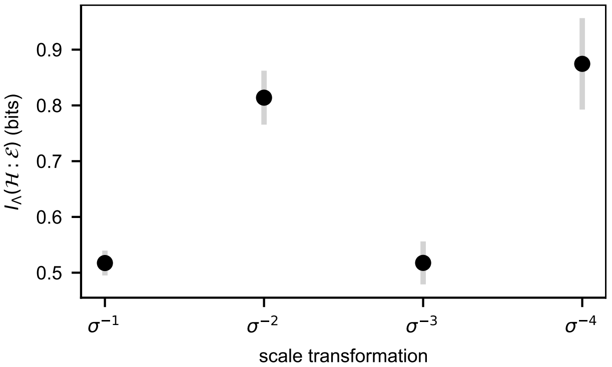

Supplementary Figure 1. Mutual information across different scale transformations. The maximal MI for the coarse graining at a 3-supervertex. For odd order rescaling transformations $\sigma^{-1,-3}$, the information attained by the compression is systematically lower compared to the even ones $\sigma^{-2,-4}$. The error bars show the standard deviation of the mutual information value over 5 independent runs of the RSMI-NE optimisation procedure.

In addition, our method finds quantitatively and qualitatively distinct behaviour at odd orders σ^{-1} and σ^{-3}. The maximal mutual information $I_\Lambda(\mathcal{H} : \mathcal{E})$ attained for the coarse graining at a 3-supervertex is non-monotonic, exhibiting, within error, two distinct values characterizing the even and odd scales (with the odd scales' information reduced to almost half), as shown in Supplementary Fig. 1.

Furthermore, the optimised coarse graining does not yield a well-defined three-state clock variable at odd scales. Indeed, for even scales the optimisation robustly yields a well-defined set of three clusters (corresponding to the three clock states) even in the distribution of pre-activations $\mathbf{\Lambda} \cdot \mathcal{V}$, while at odd scales the distribution of pre-activations lacks any such clear structure. We emphasize that this is *not* an optimization error: computationally, σ^{-4} coarse graining is a more challenging problem than σ^{-3}, due to a significantly larger number of degrees of freedom involved.

* gokmen@uchicago.edu
† flicker@physics.org
‡ maciek.kj@gmail.com

II. COARSE-GRAINING BLOCKS AND FILTERS FOR σ^{-4} DEFLATION

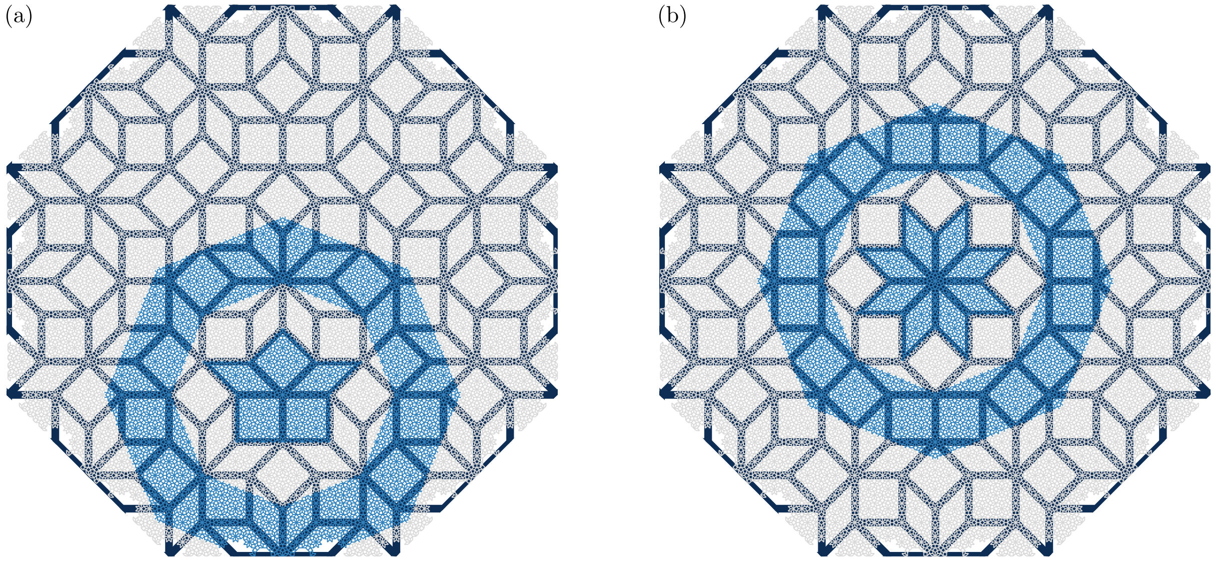

Supplementary Figure 2. Block and environment regions. Highlighted in blue are examples of the coarse-graining blocks V, and their annular environment regions E used for the 3- (a) and 8-vertices (b) at the largest scale considered (*i.e.* δ^4). The microscopic quasilattice, and the σ^{-2} super-quasilattice are shown. The centers of the 'kite' and 'star' shaped regions V are at the 8-vertices whose positions form the σ^{-4} super-quasilattice.

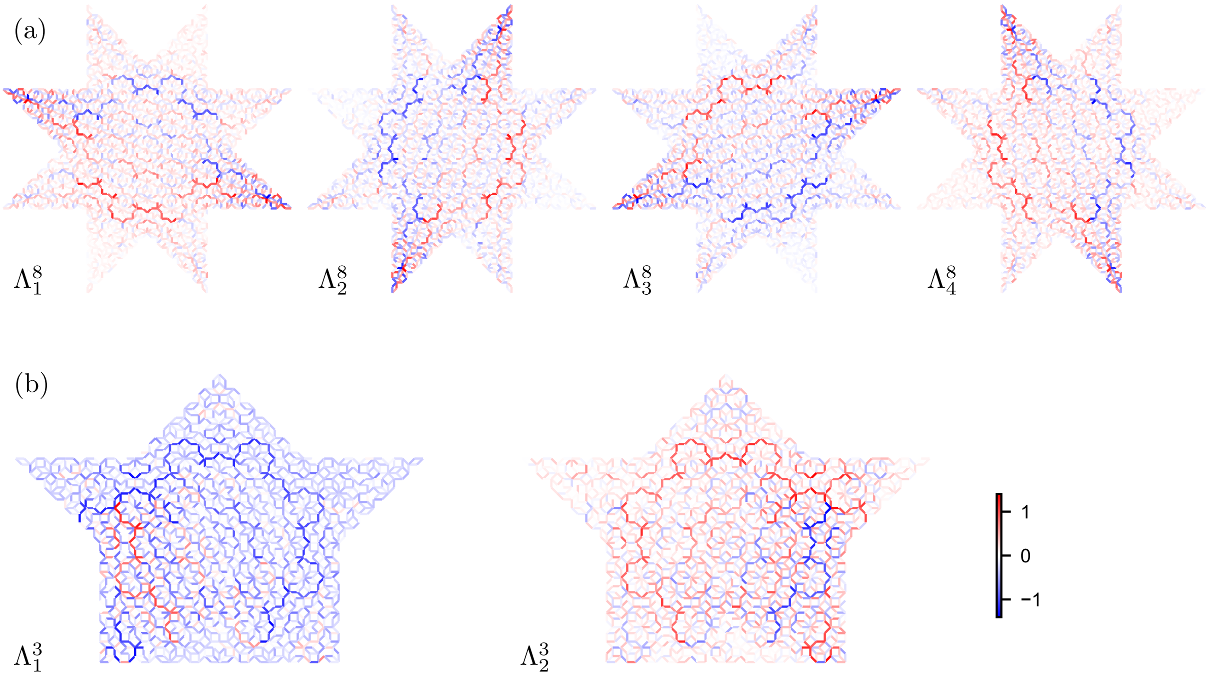

Supplementary Figure 3. Optimal σ^{-4} coarse-graining transformations. (a) 8-supervertex filters. (b) 3-supervertex filters.

III. RSMI COMPRESSION ON THE AB-TILING WITH DISTANCE BASED BLOCKS

In this section we demonstrate that our results are robust to different choices of the coarse-graining blocks. We repeat the same analysis we did for the dimer model on the AB tiling, but instead of the elementary AB tiles, we define the blocks using the graph distance. The optimal coarse-graining

transformations at the 8-vertices are shown in Supplementary Fig. 4, where length scale L_V is chosen to correspond to a σ^{-4} deflation of the AB tiling. As in the main text, we find that the emergent DOFs are discrete clock variables, with number of states equal to the connectivity of the supervertex.

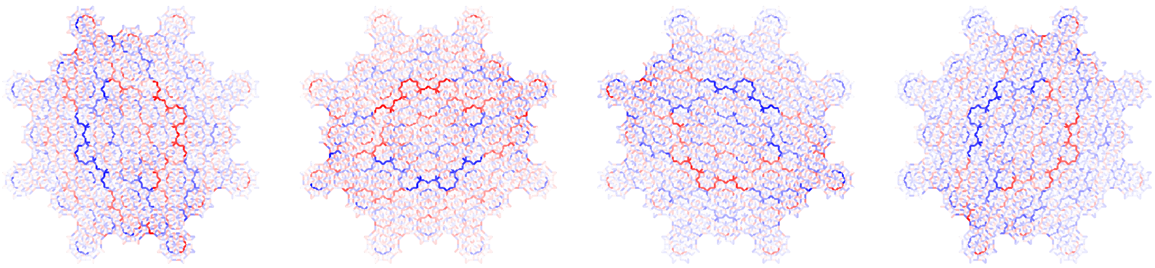

Supplementary Figure 4. Optimal σ^{-4} coarse-graining transformations at the 8-vertices of the AB tiling, where the coarse-grained blocks are defined using the graph distance instead of the elementary AB tiles in Supplementary Fig. 3.

We probe the correlations of the clock variables using conditional probabilities and find that the emergent dimer exclusion is preserved, as shown in Supplementary Fig. 5. Conditioned on an adjacent 3-clock $\mathcal{H}^3$ pointing to the centre, the probability $P(\mathcal{H}^8|\mathcal{H}^3)$ of the central 8-clock peaks at the state corresponding to the same direction. This indicates that the emergent DOFs are still paired with one and only one of their neighbours into emergent super-dimers on the edges of the super-quasilattice, as in the case of the elementary AB tiles.

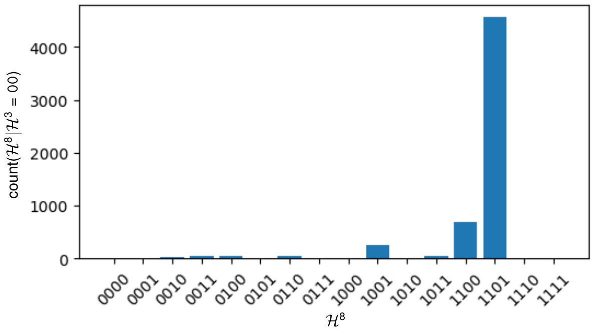

Supplementary Figure 5. The emergent dimer exclusion for resulting clock variables obtained using the coarse-grained blocks defined using graph distance. Conditioned on an adjacent 3-clock $\mathcal{H}^3$ pointing to the centre (*i.e.* $\mathcal{H}^3 = 00$), the probability $P(\mathcal{H}^8|\mathcal{H}^3)$ of the central 8-clock peaks at the state corresponding to the same direction.

IV. ANTIFERROMAGNETIC ISING MODEL ON NEARLY BIPARTITE RANDOM GRAPHS

In this section, we present an application of the RSMI-NE algorithm to frustrated antiferromagnets on random graphs, which is closely related to graph bisection and colouring problems in combinatorial optimisation and theoretical computer science [S1]. Our results demonstrate the versatility of this method across a broader class of quenched disordered systems and its applicability to more generic aperiodic graph topologies where there is no canonical choice for the block shapes.

a. Model – We consider the antiferromagnetic (AFM) Ising model on a random graph G. The Hamiltonian is given by

$$E[\boldsymbol{s}] = -J \sum_{(i,j)\in e_G} s_i s_j, \tag{S1}$$

where $J < 0$, $s_i = \pm 1$ are Ising spins, and the sum runs over all edges e_G of the graph G.

The problem of finding the ground state of this system is equivalent to dividing the set of graph nodes into two subsets, such that the number of edges across the two subsets is maximised, and those within the

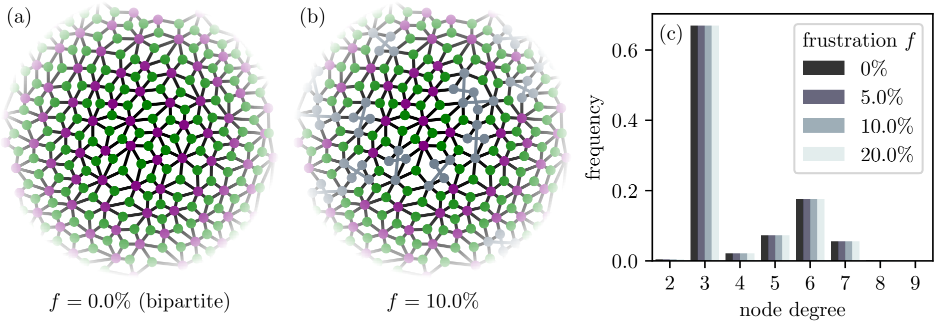

Supplementary Figure 6. Random graph ensemble with tunable frustration. (a) First, we construct random planar bipartite graphs by decorating the triangulation of a densely packed set of disks (purple nodes) with additional nodes at the triangle centres (green nodes), and placing the edges to connect the new node to the triangle corners. (b) Next, we introduce frustration by breaking the bipartite structure in a controlled way. This is done by deleting and crossing the opposite edges in a fraction f of the rhombic faces. (c) Through this process, we maintain fixed node-coordination statistics while varying the level of frustration.

subsets is minimised. The ground state energy E_0 then gives the *maximum cut* $|E_0|$. This prototypical combinatorial optimisation problem is known to be NP-complete on generic graphs [S2–S4]. However, for bipartite graphs, it reduces to the trivial two-colouring problem, where there is a maximum cut that is unique up to permutation of the partitions (or a global $\mathbb{Z}_2$ spin flip). Moving away from bipartiteness, the ground-state manifold likely becomes highly degenerate due to frustration [S1].

As a more tractable special case of this difficult problem, we narrow our focus to graphs that deviate from the bipartite structure by a small controlled amount. This case relates closely to the planted-partition model [S5], where nodes are divided into predetermined partitions, with edges placed across partitions with a small probability and within partitions with a higher probability. Planted models are useful for benchmarking improved partitioning algorithms, since the max-cut is significantly larger than the expected cut of a randomised partitioning, *cf.* generic dense random graphs [S6]. Furthermore, since the planted partition is expected to be close to the maximal cut, it is easier to verify the solution.

We will specifically consider a particular ensemble of random graphs generated via the following two-step procedure:

1. We first construct a planar graph by decorating the triangulation of a 2D random point set with additional nodes at the triangle centres, and placing the edges e_G to connect the new node to the triangle corners. This results in a graph with a (planted) bipartite structure, as shown in Supplementary Fig. 6a.

2. Following this, we introduce frustration by deliberately disrupting the bipartite structure. This involves selectively removing and crossing pairs of opposite edges within a portion f of the rhombic faces, as illustrated in Supplementary Fig. 6b. Through this process, we can adjust the level of frustration while maintaining fixed node-coordination statistics, as evidenced in Supplementary Fig. 6c. This way we can isolate the effects of frustration on the collective DOFs and their correlations without changing the effective dimensionality of the system.

On perfectly planar graphs, the duality of the max-cut problem to the route-inspection problem leads to an exact polynomial-time algorithm [S7]. Our random graph ensemble is non-planar. The above bond crossing procedure (2.) allows us to tune the hardness of the problem. In contrast to an arbitrary procedure for departing from exact-planarity, this maintains the size of the maximum cut to be significantly larger than half the number of edges. These features make this ensemble suitable to demonstrate and evaluate the performance of RSMI-NE compression in extracting relevant observables in aperiodic and amorphous geometries.

The current state-of-the-art approximation algorithm is a polynomial-time semidefinite programming method due to Goemans and Williamson. There exist other heuristic approaches such as greedy local one-exchange algorithm, which can perform well in specific ensembles. However, the convergence time of the latter often has poor scaling and it does not provide approximation guarantees as it can get stuck in local minima.

b. Results – We treat this problem as an equilibrium statistical physics model of a magnet, exhibiting critical phenomena. For the bipartite case ($f = 0\%$), it is expected that this system undergoes a second-order phase transition into a long-range ordered AFM phase at a sufficiently low temperature. It is then

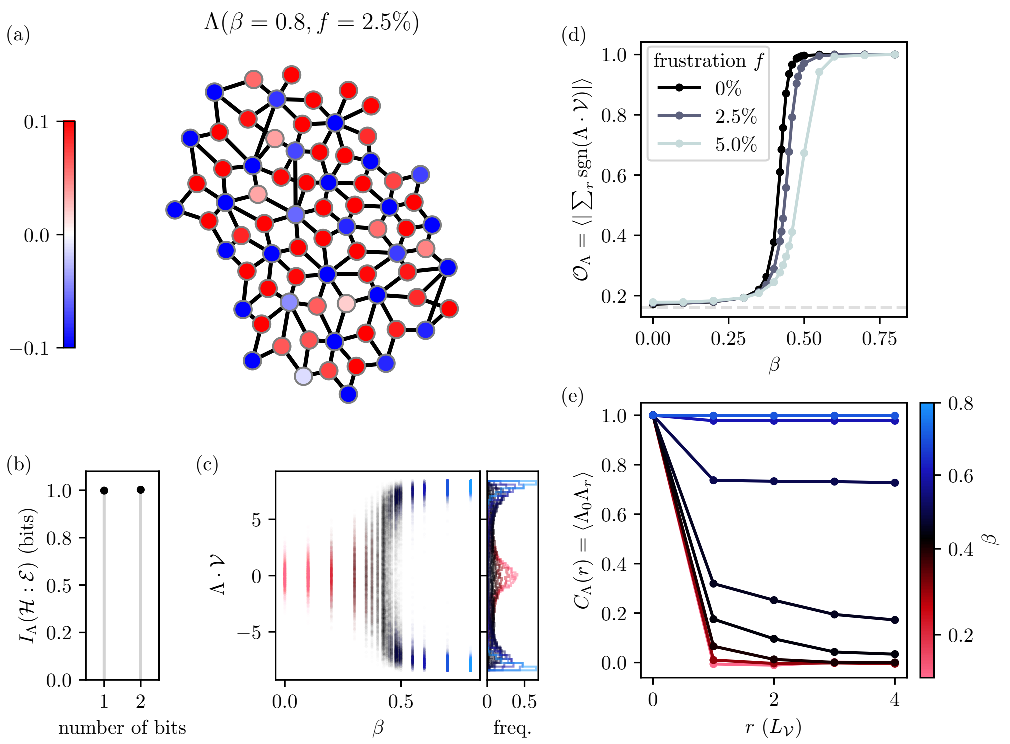

Supplementary Figure 7. (a) The optimal coarse-graining filter Λ at frustration $f = 2.5\%$ and inverse temperature $\beta = 0.8$. (b) We use a 1-bit coarse-grained variable $\mathcal{H} = \mathrm{sgn}(\Lambda \cdot \mathcal{V})$ as the mutual information value saturates using a single filter component. (c) The distribution of the pre-activation $\Lambda \cdot \mathcal{V}$ transitions from unimodal to bimodal as a function of β, indicating spontaneous symmetry breaking. (d) The individually optimised local coarse-graining transformations can be combined into a global order parameter $\mathcal{O}_\Lambda$ for finite f. (e) The correlation function of the coarse-grained variable $\mathcal{H}$ exhibits long-range order at low-temperature for finite frustration (here $f = 2.5\%$).

natural to ask whether this long-range order persists under finite frustration. Answering this question requires constructing an AFM order parameter on random graphs: a task which becomes challenging when the bipartitioning is neither known nor unique.

To address these questions, we apply the RSMI-NE compression on Monte Carlo configurations generated using the Wolff cluster algorithm [S8]. We construct the block $\mathcal{V} = [s_i]_{i\,\in V}$ and the environment $\mathcal{E} = [s_i]_{i\,\in E}$ using sets of nodes V, E partitioned via the graph distance. In particular we set $L_V = 5$, $L_B = 8$, and $L_{E_{\mathrm{out}}} - L_{E_{\mathrm{in}}} = 8$. Like in Fig. 3 in the main text, the number of filters is determined by the plateauing of mutual information, which, in this case, is 1 bit (Supplementary Fig. 7b).

The optimal coarse-graining filter successfully identifies the correct planted bipartitioning (Supplementary Fig. 7a) on the block $\mathcal{V}$ at frustration $f \leq 7.5\%$. (We benchmark the bipartitioning of the RSMI-compression against other standard algorithms below.) In turn, the emergent DOF $\mathcal{H} = \mathrm{sgn}(\Lambda \cdot \mathcal{V})$ is a local order parameter for the AFM order. This can be seen in Supplementary Fig. 7c, where the distribution of the pre-activation $\Lambda \cdot \mathcal{V}$ transitions from unimodal to bimodal as a function β.

To check if the long-range order persists under finite frustration, we need to construct a global order parameter. We can do this by combining the individually optimised local coarse-graining transformations at blocks $\mathcal{V}^i$ that span the entire graph:

$$\mathcal{O}_\Lambda = \left\langle \left\| \sum_i \mathrm{sgn}(\Lambda^i \cdot \mathcal{V}^i) \right\| \right\rangle = \left\langle \left\| \sum_i \mathcal{H}^i \right\| \right\rangle, \tag{S2}$$

Since the mutual information is invariant under a sign flip $\mathcal{H} \mapsto -\mathcal{H}$, constructing $\mathcal{O}$ requires fixing the gauge for independently trained Λ^i. We do this by a ‘stitching’ procedure, where we take slightly overlapped regions $\mathcal{V}^i$ and match the sign of the filters Λ^i in the overlapping region by flipping the sign of the entire filter, when necessary.

Computing $\mathcal{O}_\Lambda(\beta)$ (Supplementary Fig. 7d), we find that the system perfectly ‘magnetises’ at low temperatures for $f \leq 7.5\%$, while the sole effect of frustration is to shift the critical temperature to lower

values. Finally, we can probe the correlation function of the coarse-grained variable $\mathcal{H}$, which exhibits long-range order at $\beta > 0.5$ for finite frustration, as shown in Supplementary Fig. 7e.

Combined with the above mutual information gauge stitching procedure, the RSMI-NE algorithm thus assisted us in constructing a global order parameter for the antiferromagnetic phase on random graphs. This enabled us to study the effects of frustration on long-range order without prior knowledge of the bipartitioning, and conclude that finite but small frustration $f \leq 7.5\%$ is irrelevant for large-scale physics.

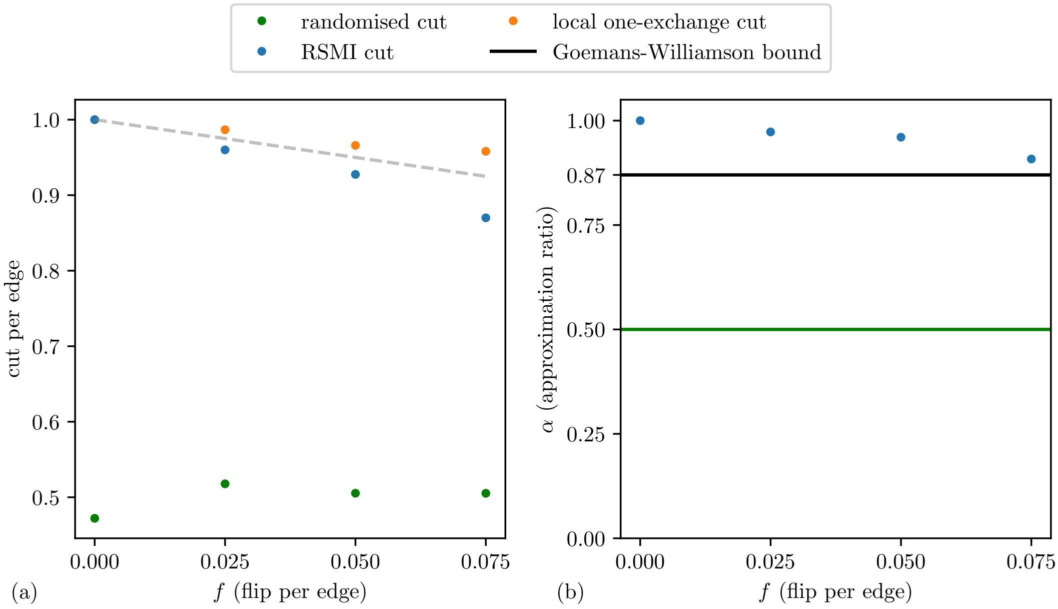

Supplementary Figure 8. Benchmarking RSMI-NE in the max-cut problem. (a) The size of the cut (per edge) for different algorithms. The RSMI-NE cut (blue) gets close to the optimal solution found by the greedy local one-exchange strategy (orange). The max-cut is significantly larger than the randomised cut (green) of 0.5 per edge. The gray dashed line is $1 - f$. (b) Approximation ratio for RSMI-NE is higher than the performance guarantee of $\alpha \geq 0.87$ for the Goemans-Williamson algorithm (solid black line).

c. Benchmarking. Since max-cut is NP-hard, exact algorithms, such as branch and bound, are infeasible for the graphs we consider with several hundreds of nodes. Moreover, this problem is also APX-hard [S9], *i.e.* it does not have a polynomial-time approximation scheme that is guaranteed to converge to the global extrema. Nevertheless, there exist approximation algorithms that are guaranteed find a cut that is at least a certain proportion of the optimal value.

A commonly used metric to measure the performance of an approximation scheme is the *approximation ratio*

$$\alpha(E) = \frac{E}{E_0}, \tag{S3}$$

so that $\alpha = 1$ when the cut is indeed maximal.

We first provide a comparison of RSMI-NE with randomised partitioning and greedy one-exchange local search algorithms. For this benchmarking, we consider instances from the above random graph ensemble in a range of values for $0\% < f < 7.5\%$, each graph consisting of 1084 nodes in total. As shown in Supplementary Fig. 8a, the RSMI cut fraction is comparable to the one-exchange algorithm, which is expected to perform well in the regime close to exact bipartiteness and planarity (in the shown instances, it indeed finds the global optimum). Note however, that the convergence time of iterated local search exceeded the runtime of RSMI-NE significantly and for the graphs we considered (see Supplementary Fig. 9).

There are a few factors to consider regarding the scaling of RSMI-NE. Firstly, the runtime is mostly dominated by the deep neural network critic function f (Eq. 10), whose task is to estimate the mutual information. Since the environment $\mathcal{E}$ comprises complementary set of nodes to $\mathcal{V}$, the forward passes of f involve the combined $(\mathcal{V}, \mathcal{E})$ graphs that are always roughly the same size. Therefore, this part of the computation should not scale with the number of nodes in $\mathcal{V}$. The main contributor of the scaling is the increasing number of training iterations till reaching 1-bit, followed by a weaker contribution from the forward passes of the compression map $\Lambda \cdot \mathcal{V}$, which is a linear neural network (computationally

much lighter than f). Note also that the runtime of the Monte Carlo algorithm is included to RSMI-NE in Supplementary Fig. 9. While the Wolff algorithm has linear scaling with the number of nodes, we obtained the $(\mathcal{V}, \mathcal{E})$ configurations from the same one large graph of 1804 nodes. Therefore, the MC runtime amounts to a constant offset of 2.36 seconds for generating 10k samples on this graph.

One major drawback of the one-exchange algorithm is the lack of a formal guarantee to find the globally optimal solution as it can get stuck in local minima. A reference benchmark *with* an approximation guarantee is the Goemans-Williamson (GW) algorithm [S6]. It has the best known approximation guarantee, $\alpha \geq 0.87$. We observed that the approximation ratio of RSMI exceeds the worst-case scenario of the GW algorithm (Supplementary Fig. 8b). Notice though, that the performance of RSMI-NE degrades with increasing f, and eventually drops below the GW bound.

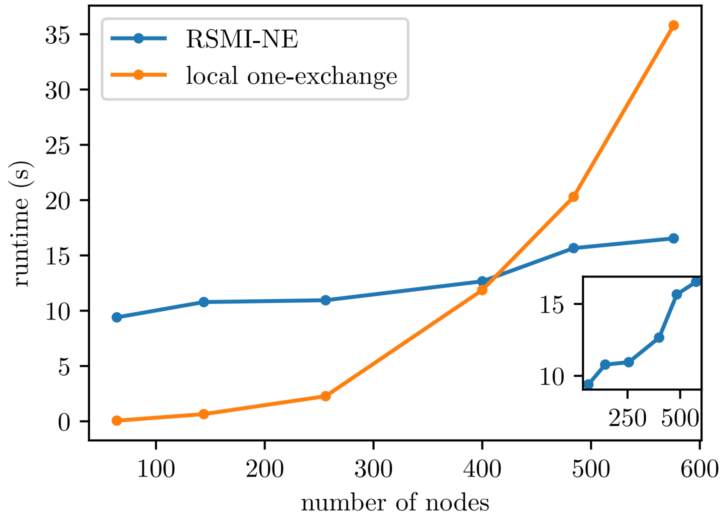

Supplementary Figure 9. Runtime scaling of the local one-exchange algorithm and the RSMI-NE algorithm with the number of nodes in the graph. The local search algorithm runs a greedy one-exchange strategy until no improvement can be made. The RSMI-NE algorithm is ran until it achieves 1-bit of mutual information, which signals when the compression map captures the long-range order. The sudden increase in runtime for RSMI-NE around 500 nodes (see the inset) is due to the increased number of epochs to reach 1-bit.

We emphasise that RSMI-NE had not been hard-coded to solve the max-cut problem, but it has *identified* this as the most relevant question, and extracted the AFM order parameter. While tuning up f, one needs to consider the possibility that this system undergoes a qualitative change, whereby antiferromagnetism is destroyed. In this case, the RSMI compression map may start to track a *new* slow observable, whose mixing with the AFM order in the intermediate regime might be the reason for the degrading the max-cut performance. These questions concerning the extended phase diagram of Eq. S1 go beyond the scope of this appendix and we leave this interesting possibility for a future study.

Since the RSMI-NE compression selects the bipartitioning as the correct relevant observable, the present results suggest that one can indeed directly use one of the existing bipartitioning algorithms (*e.g.* the Goemans-Williamson algorithm, see below) as a *computational order paremeter* [S10] to detect the long-range ordering in this system, also without employing any coarse-graining procedure. In Supplementary Fig. 10a, we plot the temperature dependence of such an AFM order parameter constructed according to the one-exchange bipartitioning ($f = 5\%$):

$$m = \sum_{i \in \text{set1}} s_i - \sum_{i \in \text{set2}} s_i. \tag{S4}$$

We also provide a coarse grained version of this order parameter in Supplementary Fig. 10b, defined analogously to Eq. S2. Again we construct Kadanoff block spins on blocks of radius $L_V = 5$, which leads to a 5×5 coarse grained lattice. Note that the dashed line indicates the exact value for the non-vanishing absolute magnetisation density of a perfect paramagnet on a 5×5 lattice. Both versions indicate a phase transition around $\beta \approx 0.45$. It is unsurprising that the behaviour of this computational observable is almost identical to Fig. 7d, as the RSMI-NE achieved an approximation ratio $\alpha \approx 0.95$ for the max-cut (Supplementary Fig. 8b).

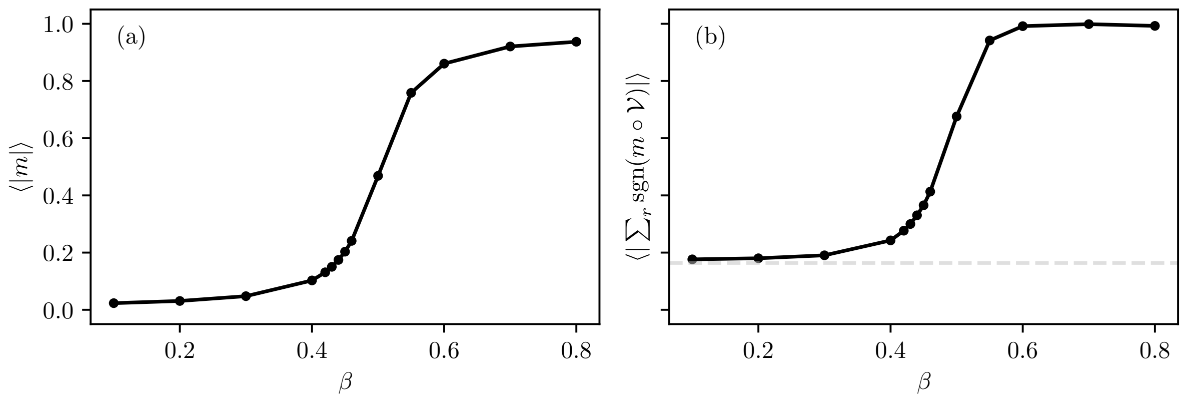

Supplementary Figure 10. (a) Computational AFM order parameter constructed via the one-exchange local greedy search algorithm. (b) Coarse grained version. The temperature dependence is shown for the same $f = 5\%$ random graph as for Supplementary Fig. 7d.

SUPPLEMENTARY REFERENCES

[S1] M. Mézard and G. Parisi, Mean-field theory of randomly frustrated systems with finite connectivity, Europhysics Letters **3**, 1067 (1987).

[S2] M. Garey, D. Johnson, and L. Stockmeyer, Some simplified np-complete graph problems, Theoretical Computer Science **1**, 237 (1976).

[S3] M. R. Garey and D. S. Johnson, *Computers and Intractability: A Guide to the Theory of NP-Completeness (Series of Books in the Mathematical Sciences)*, first edition ed. (W. H. Freeman, 1979).

[S4] R. M. Karp, Reducibility among combinatorial problems, in *Complexity of Computer Computations: Proceedings of a symposium on the Complexity of Computer Computations, held March 20–22, 1972, at the IBM Thomas J. Watson Research Center, Yorktown Heights, New York, and sponsored by the Office of Naval Research, Mathematics Program, IBM World Trade Corporation, and the IBM Research Mathematical Sciences Department*, edited by R. E. Miller, J. W. Thatcher, and J. D. Bohlinger (Springer US, Boston, MA, 1972) pp. 85–103.

[S5] B. Bollobás and A. D. Scott, Max cut for random graphs with a planted partition, Combinatorics, Probability and Computing **13**, 451–474 (2004).

[S6] M. X. Goemans and D. P. Williamson, Improved approximation algorithms for maximum cut and satisfiability problems using semidefinite programming, J. ACM **42**, 1115–1145 (1995).

[S7] F. Hadlock, Finding a maximum cut of a planar graph in polynomial time, SIAM Journal on Computing **4**, 221 (1975), https://doi.org/10.1137/0204019.

[S8] U. Wolff, Collective monte carlo updating for spin systems, Phys. Rev. Lett. **62**, 361 (1989).

[S9] C. H. Papadimitriou and M. Yannakakis, Optimization, approximation, and complexity classes, Journal of Computer and System Sciences **43**, 425 (1991).

[S10] Z. Weinstein, J. A. Ahmad, D. Podolsky, and E. Altman, Computational phase transitions in two-dimensional antiferromagnetic melting (2024), arXiv:2407.18405 [cond-mat.stat-mech].
